# 3a,11b-Dihy­droxy-3a,11b-dihydro-1*H*-imidazo[4,5-*f*][1,10]phenanthroline-2(3*H*)-thione

**DOI:** 10.1107/S1600536811053967

**Published:** 2011-12-21

**Authors:** Hua Wang, Peng Mei, Wen-Yi Chu, Zhi-Zhong Sun, Yan-Jun Hou

**Affiliations:** a College of Chemistry and Materials Science, Heilongjiang University, Harbin 150080, People’s Republic of China

## Abstract

The title compound, C_13_H_10_N_4_O_2_S, was prepared through a cyclization reaction of 1,10-phenanthroline-5,6-dione and thio­urea. The dihedral angle between the pyridine rings is 8.22 (2)°. In the crystal, mol­ecules are connected by N—H⋯O, O—H⋯N, N—H⋯S and O—H⋯S hydrogen bonds, forming a three-dimensional network.

## Related literature

For related structures, see: Liu *et al.* (2008 ▶); Wang *et al.* (2011 ▶); Cong *et al.* (2009 ▶).
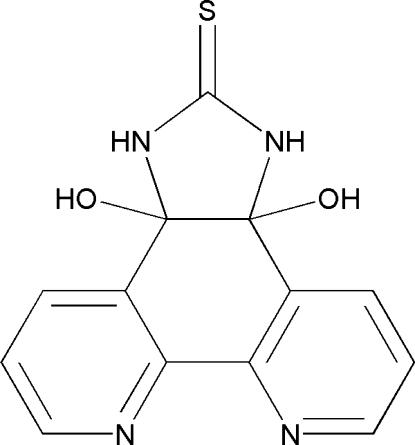


## Experimental

### 

#### Crystal data

C_13_H_10_N_4_O_2_S
*M* *_r_* = 286.31
Monoclinic, 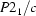

*a* = 11.259 (4) Å
*b* = 12.815 (4) Å
*c* = 8.565 (3) Å
β = 100.382 (5)°
*V* = 1215.6 (7) Å^3^
*Z* = 4
Mo *K*α radiation
μ = 0.27 mm^−1^
*T* = 293 K
0.18 × 0.14 × 0.12 mm

#### Data collection

Bruker SMART APEXII CCD detector diffractometer
Absorption correction: multi-scan (*SADABS*; Sheldrick, 1996 ▶) *T*_min_ = 0.952, *T*_max_ = 0.968
9690 measured reflections
3035 independent reflections
1325 reflections with *I* > 2σ(*I*)
*R*_int_ = 0.107

#### Refinement

*R*[*F* ^2^ > 2σ(*F* ^2^)] = 0.061
*wR*(*F* ^2^) = 0.161
*S* = 0.94
3035 reflections
191 parameters
H atoms treated by a mixture of independent and constrained refinement
Δρ_max_ = 0.38 e Å^−3^
Δρ_min_ = −0.30 e Å^−3^

### 

Data collection: *APEX2* (Bruker, 2004 ▶); cell refinement: *SAINT* (Bruker, 2004 ▶); data reduction: *SAINT*; program(s) used to solve structure: *SHELXS97* (Sheldrick, 2008 ▶); program(s) used to refine structure: *SHELXL97* (Sheldrick, 2008 ▶); molecular graphics: *SHELXTL* (Sheldrick, 2008 ▶); software used to prepare material for publication: *publCIF* (Westrip, 2010 ▶).

## Supplementary Material

Crystal structure: contains datablock(s) I, global. DOI: 10.1107/S1600536811053967/bq2329sup1.cif

Supplementary material file. DOI: 10.1107/S1600536811053967/bq2329Isup2.cdx

Structure factors: contains datablock(s) I. DOI: 10.1107/S1600536811053967/bq2329Isup3.hkl

Supplementary material file. DOI: 10.1107/S1600536811053967/bq2329Isup4.cml

Additional supplementary materials:  crystallographic information; 3D view; checkCIF report

## Figures and Tables

**Table 1:** Hydrogen-bond geometry (Å, °)

*D*—H⋯*A*	*D*—H	H⋯*A*	*D*⋯*A*	*D*—H⋯*A*
N3—H3*N*⋯O1^i^	0.93 (4)	2.08 (4)	2.980 (4)	162 (3)
O2—H2*O*⋯S1^ii^	0.82	2.49	3.276 (3)	160
N4—H4*N*⋯S1^iii^	0.90 (4)	2.48 (4)	3.365 (4)	166 (3)
O1—H1*O*⋯N1^iv^	0.82	2.33	2.930 (4)	130
O1—H1*O*⋯N2^iv^	0.82	2.26	3.032 (4)	158

Symmetry codes: (i) 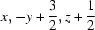; (ii) 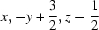; (iii) 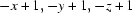; (iv) 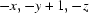.

**Fig. 1.**
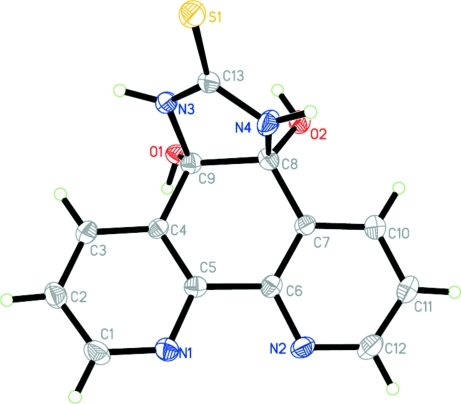
The molecular structure of the title compound I, with displacement ellipsoids drawn at the 50% probability level.

**Fig. 2.**
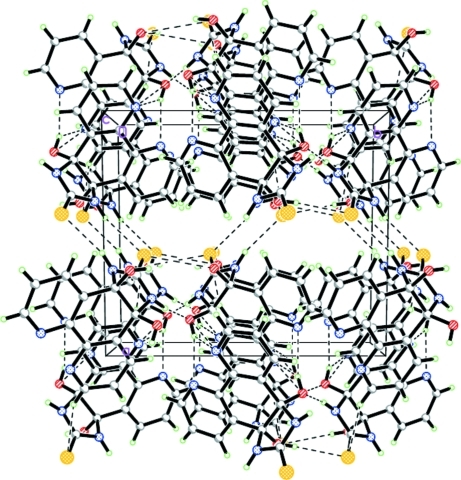
Part of the packing of the title compound, viewed down the c-axis. Dashed lines indicate hydrogen bonds.

## supplementary crystallographic information

### Comment

Considerable interest have been paid to the reactions of various metal salts with multi-carboxylate ligands and 1,10-phenanthroline-5,6-dione and the influence of the reaction pH on the structure of the resultant complexes (Liu *et al.*, 2008; Wang *et al.*, 2011; Cong *et al.*, 2009). We prepare 3a,11*b*-dihydroxy-3,3a-dihydro-1*H*-imidazo [4,5-*f*][1,10]phenanthroline-2(11bH)-thione as a precursor of 1,10-phenanthroline-5,6-dione for precise control of the reaction pH.

As shown in Fig. 1, The dihedral angle between the pyridine rings (C1-C5/N1) (C6-C7/C10-C12/N2) is 8.22 (2)°. The neighboring molecules are connected by N-H···O, O-H···N, N-H···Si and O-H···Si hydrogen bonds to form an infinite three-dimensional network (Table 1. and Fig. 2).

### Experimental

40 ml 98% H_2_SO_4_ and 20 ml 69% HNO_3_ were mixed in a flask and cooled to 273 K. Then A mixture of 1,10-phenanthroline (4 g, 22.2 mmol) and KBr (4 g, 33.6 mmol) was slowly added while keeping the temperature below 279 K. The resulting solution was refluxed for 4 hr and finally cooled to room temperature. The contents of the flask were poured onto 100 g crushed ice and neutralized with 40% sodium hydroxide solution. The yellow precipitate of 1,10-phenanthroline-5,6-dione was collected by filtration and washed with water. The filtrate was extracted with EtOAc, the organic phase was dried over magnesium sulfate and the solvent was evaporated off under vacuum. All of the crude product was then recrystallized from 100 mL EtOH to give 2.6 g of 1,10-phenanthroline-5,6-dione as yellow needles. The product of the reaction mentioned above was reacted with thiourea (13 g, 217 mmol) in 50 ml methanol for 5 hr under reflux. After cooling, the precipitated product was separated and recrystallized from EtOH to give 2.1 g (63%) of 1,11*b*-dihydro-3a,11*b*-dihydroxy-1*H*-imidazo[4,5-*f*][1,10] phenanthroline-2(11bH)-thione as white powder. Crystals suitable for single-crystal X-ray diffraction were obtained by recrystallization from methanol at room temperature in a total yield of 24%. Anal. Calcd. for C_13_H_10_N_4_O_2_S: C, 54.54; H, 3.52; N, 19.57. Found(%):*C*, 54.60; H, 3.58; N, 19.66. IR(KBr) ^1^H NMR (400 MHz, DMSO-d^6^): 9.47 (s, 2H), 8.71 (dd, *J* = 4.6, 1.7 Hz, 2H), 8.23 (dd, *J* = 7.9, 1.7 Hz, 2H), 7.53 (dd, *J* = 7.9, 4.6 Hz, 2H), 6.83 (s, 2H), 3.31 (s, 2H).

### Refinement

H atoms bound to C atoms were placed in calculated positions and treated as riding on their parent atoms with C—H = 0.93 Å (aromatic C), and with *U*_iso_(H) = 1.2Ueq(C). H atoms bound to O atoms were placed in calculated positions and treated as riding on their parent atoms, with O—H = 0.82 Å and with *U*_iso_(H) = 1.5Ueq(O). H atoms bound to N atoms were located in the difference-Fourier map and refined isotropically.

### Crystal data

**Table:**

C_13_H_10_N_4_O_2_S	*F*(000) = 592
*M**_r_* = 286.31	*D*_x_ = 1.564 Mg m^−^^3^
Monoclinic, *P*2_1_/*c*	Mo *K*α radiation, λ = 0.71073 Å
Hall symbol: -P 2ybc	Cell parameters from 512 reflections
*a* = 11.259 (4) Å	θ = 2.4–19.3°
*b* = 12.815 (4) Å	µ = 0.27 mm^−^^1^
*c* = 8.565 (3) Å	*T* = 293 K
β = 100.382 (5)°	Block, colorless
*V* = 1215.6 (7) Å^3^	0.18 × 0.14 × 0.12 mm
*Z* = 4	

### Data collection

**Table:**

Bruker SMART APEXII CCD detector diffractometer	3035 independent reflections
Radiation source: fine-focus sealed tube	1325 reflections with *I* > 2σ(*I*)
graphite	*R*_int_ = 0.107
phi and ω scans	θ_max_ = 28.3°, θ_min_ = 2.4°
Absorption correction: multi-scan (*SADABS*; Sheldrick, 1996)	*h* = −14→15
*T*_min_ = 0.952, *T*_max_ = 0.968	*k* = −17→10
9690 measured reflections	*l* = −11→10

### Refinement

**Table:**

Refinement on *F*^2^	Primary atom site location: structure-invariant direct methods
Least-squares matrix: full	Secondary atom site location: difference Fourier map
*R*[*F*^2^ > 2σ(*F*^2^)] = 0.061	Hydrogen site location: inferred from neighbouring sites
*wR*(*F*^2^) = 0.161	H atoms treated by a mixture of independent and constrained refinement
*S* = 0.94	*w* = 1/[σ^2^(*F*_o_^2^) + (0.0587*P*)^2^] where *P* = (*F*_o_^2^ + 2*F*_c_^2^)/3
3035 reflections	(Δ/σ)_max_ < 0.001
191 parameters	Δρ_max_ = 0.38 e Å^−^^3^
0 restraints	Δρ_min_ = −0.30 e Å^−^^3^

### Special details

**Table:**

Geometry. All e.s.d.'s (except the e.s.d. in the dihedral angle between two l.s. planes) are estimated using the full covariance matrix. The cell e.s.d.'s are taken into account individually in the estimation of e.s.d.'s in distances, angles and torsion angles; correlations between e.s.d.'s in cell parameters are only used when they are defined by crystal symmetry. An approximate (isotropic) treatment of cell e.s.d.'s is used for estimating e.s.d.'s involving l.s. planes.
Refinement. Refinement of *F*^2^ against ALL reflections. The weighted *R*-factor *wR* and goodness of fit *S* are based on *F*^2^, conventional *R*-factors *R* are based on *F*, with *F* set to zero for negative *F*^2^. The threshold expression of *F*^2^ > σ(*F*^2^) is used only for calculating *R*-factors(gt) *etc*. and is not relevant to the choice of reflections for refinement. *R*-factors based on *F*^2^ are statistically about twice as large as those based on *F*, and *R*- factors based on ALL data will be even larger.

### Fractional atomic coordinates and isotropic or equivalent isotropic displacement parameters (Å^2^)

**Table:**

	*x*	*y*	*z*	*U*_iso_*/*U*_eq_	
S1	0.41534 (9)	0.63611 (8)	0.62203 (12)	0.0424 (3)	
O1	0.1477 (2)	0.70072 (19)	0.1051 (3)	0.0370 (7)	
H1O	0.0819	0.6850	0.0525	0.055*	
O2	0.3623 (2)	0.6130 (2)	0.0847 (3)	0.0452 (7)	
H2O	0.3702	0.6733	0.1168	0.068*	
N1	−0.0346 (3)	0.3969 (2)	0.1943 (4)	0.0387 (8)	
N2	0.1205 (3)	0.3145 (2)	0.0259 (4)	0.0378 (8)	
N3	0.2442 (3)	0.6720 (3)	0.3648 (4)	0.0351 (8)	
N4	0.3596 (3)	0.5401 (3)	0.3380 (4)	0.0412 (9)	
C5	0.0627 (3)	0.4558 (3)	0.1826 (4)	0.0301 (8)	
C9	0.1854 (3)	0.6227 (3)	0.2162 (4)	0.0302 (8)	
C4	0.0789 (3)	0.5553 (3)	0.2417 (4)	0.0269 (8)	
C7	0.2619 (3)	0.4546 (3)	0.0946 (4)	0.0310 (9)	
C6	0.1524 (3)	0.4060 (3)	0.0979 (4)	0.0305 (9)	
C8	0.2935 (3)	0.5569 (3)	0.1767 (4)	0.0337 (9)	
C3	−0.0097 (3)	0.5981 (3)	0.3175 (4)	0.0357 (9)	
H3	−0.0016	0.6652	0.3591	0.043*	
C13	0.3384 (3)	0.6167 (3)	0.4379 (4)	0.0326 (9)	
C10	0.3448 (3)	0.4034 (3)	0.0190 (5)	0.0447 (11)	
H10	0.4196	0.4331	0.0152	0.054*	
C2	−0.1093 (3)	0.5385 (3)	0.3291 (4)	0.0405 (10)	
H2A	−0.1703	0.5651	0.3777	0.049*	
C1	−0.1175 (3)	0.4392 (3)	0.2681 (5)	0.0428 (10)	
H1A	−0.1846	0.3992	0.2788	0.051*	
C12	0.2019 (4)	0.2682 (3)	−0.0466 (5)	0.0458 (11)	
H12	0.1808	0.2051	−0.0978	0.055*	
C11	0.3136 (4)	0.3072 (3)	−0.0505 (5)	0.0503 (11)	
H11	0.3680	0.2701	−0.0988	0.060*	
H4N	0.428 (3)	0.503 (3)	0.356 (4)	0.043 (11)*	
H3N	0.203 (3)	0.717 (3)	0.421 (4)	0.047 (12)*	

### Atomic displacement parameters (Å^2^)

**Table:**

	*U*^11^	*U*^22^	*U*^33^	*U*^12^	*U*^13^	*U*^23^
S1	0.0378 (5)	0.0448 (7)	0.0423 (6)	0.0060 (5)	0.0008 (4)	−0.0064 (5)
O1	0.0349 (14)	0.0285 (16)	0.0458 (16)	−0.0037 (12)	0.0027 (12)	0.0078 (13)
O2	0.0439 (16)	0.0358 (17)	0.0624 (19)	−0.0067 (14)	0.0269 (14)	−0.0091 (14)
N1	0.0371 (18)	0.032 (2)	0.049 (2)	−0.0024 (15)	0.0144 (16)	0.0008 (15)
N2	0.0457 (19)	0.0283 (19)	0.0409 (19)	−0.0063 (15)	0.0117 (15)	−0.0059 (15)
N3	0.0282 (17)	0.035 (2)	0.0402 (19)	0.0029 (15)	0.0013 (14)	−0.0086 (16)
N4	0.0374 (19)	0.039 (2)	0.043 (2)	0.0132 (17)	−0.0035 (16)	−0.0121 (17)
C5	0.0320 (19)	0.027 (2)	0.0303 (19)	−0.0002 (17)	0.0027 (15)	0.0040 (17)
C9	0.0324 (19)	0.025 (2)	0.0322 (19)	0.0015 (16)	0.0043 (16)	0.0023 (17)
C4	0.0304 (19)	0.022 (2)	0.0281 (19)	0.0016 (16)	0.0040 (15)	0.0004 (16)
C7	0.031 (2)	0.028 (2)	0.033 (2)	−0.0005 (16)	0.0037 (16)	−0.0034 (17)
C6	0.034 (2)	0.029 (2)	0.0282 (19)	0.0040 (17)	0.0030 (16)	0.0005 (16)
C8	0.0274 (19)	0.036 (2)	0.038 (2)	0.0007 (17)	0.0055 (16)	−0.0004 (19)
C3	0.037 (2)	0.029 (2)	0.042 (2)	0.0031 (17)	0.0081 (17)	−0.0049 (18)
C13	0.0277 (19)	0.029 (2)	0.041 (2)	−0.0016 (16)	0.0061 (17)	−0.0037 (18)
C10	0.037 (2)	0.044 (3)	0.056 (3)	−0.0013 (19)	0.016 (2)	−0.011 (2)
C2	0.032 (2)	0.040 (3)	0.051 (3)	0.0043 (19)	0.0135 (18)	0.002 (2)
C1	0.038 (2)	0.039 (3)	0.054 (3)	−0.0058 (19)	0.0148 (19)	0.009 (2)
C12	0.061 (3)	0.032 (3)	0.047 (2)	−0.001 (2)	0.016 (2)	−0.008 (2)
C11	0.053 (3)	0.040 (3)	0.062 (3)	0.005 (2)	0.019 (2)	−0.014 (2)

### Geometric parameters (Å, °)

**Table:**

S1—C13	1.676 (4)	C9—C4	1.524 (5)
O1—C9	1.393 (4)	C9—C8	1.568 (5)
O1—H1O	0.8200	C4—C3	1.397 (5)
O2—C8	1.399 (4)	C7—C6	1.387 (5)
O2—H2O	0.8200	C7—C10	1.392 (5)
N1—C1	1.333 (4)	C7—C8	1.499 (5)
N1—C5	1.349 (4)	C3—C2	1.375 (5)
N2—C12	1.335 (5)	C3—H3	0.9300
N2—C6	1.343 (4)	C10—C11	1.386 (5)
N3—C13	1.334 (4)	C10—H10	0.9300
N3—C9	1.467 (4)	C2—C1	1.372 (5)
N3—H3N	0.93 (4)	C2—H2A	0.9300
N4—C13	1.351 (4)	C1—H1A	0.9300
N4—C8	1.463 (5)	C12—C11	1.360 (5)
N4—H4N	0.90 (4)	C12—H12	0.9300
C5—C4	1.372 (5)	C11—H11	0.9300
C5—C6	1.489 (5)		
			
C9—O1—H1O	109.5	O2—C8—N4	111.6 (3)
C8—O2—H2O	109.5	O2—C8—C7	107.0 (3)
C1—N1—C5	117.1 (3)	N4—C8—C7	110.6 (3)
C12—N2—C6	116.9 (3)	O2—C8—C9	112.1 (3)
C13—N3—C9	112.0 (3)	N4—C8—C9	99.1 (3)
C13—N3—H3N	121 (2)	C7—C8—C9	116.4 (3)
C9—N3—H3N	122 (2)	C2—C3—C4	118.4 (3)
C13—N4—C8	111.9 (3)	C2—C3—H3	120.8
C13—N4—H4N	122 (2)	C4—C3—H3	120.8
C8—N4—H4N	121 (2)	N3—C13—N4	107.8 (3)
N1—C5—C4	123.3 (3)	N3—C13—S1	126.6 (3)
N1—C5—C6	115.3 (3)	N4—C13—S1	125.7 (3)
C4—C5—C6	121.4 (3)	C11—C10—C7	118.7 (4)
O1—C9—N3	108.6 (3)	C11—C10—H10	120.7
O1—C9—C4	110.7 (3)	C7—C10—H10	120.7
N3—C9—C4	111.3 (3)	C1—C2—C3	119.2 (4)
O1—C9—C8	113.0 (3)	C1—C2—H2A	120.4
N3—C9—C8	99.9 (3)	C3—C2—H2A	120.4
C4—C9—C8	112.8 (3)	N1—C1—C2	123.5 (4)
C5—C4—C3	118.5 (3)	N1—C1—H1A	118.2
C5—C4—C9	122.0 (3)	C2—C1—H1A	118.2
C3—C4—C9	119.4 (3)	N2—C12—C11	124.3 (4)
C6—C7—C10	118.1 (3)	N2—C12—H12	117.8
C6—C7—C8	121.3 (3)	C11—C12—H12	117.8
C10—C7—C8	120.5 (3)	C12—C11—C10	118.7 (4)
N2—C6—C7	123.2 (3)	C12—C11—H11	120.6
N2—C6—C5	116.7 (3)	C10—C11—H11	120.6
C7—C6—C5	120.1 (3)		
			
C1—N1—C5—C4	−0.3 (5)	C10—C7—C8—O2	37.4 (4)
C1—N1—C5—C6	−179.4 (3)	C6—C7—C8—N4	92.5 (4)
C13—N3—C9—O1	−142.9 (3)	C10—C7—C8—N4	−84.4 (4)
C13—N3—C9—C4	95.0 (3)	C6—C7—C8—C9	−19.5 (5)
C13—N3—C9—C8	−24.4 (4)	C10—C7—C8—C9	163.6 (3)
N1—C5—C4—C3	−0.1 (5)	O1—C9—C8—O2	25.6 (4)
C6—C5—C4—C3	178.9 (3)	N3—C9—C8—O2	−89.6 (3)
N1—C5—C4—C9	−175.5 (3)	C4—C9—C8—O2	152.1 (3)
C6—C5—C4—C9	3.5 (5)	O1—C9—C8—N4	143.4 (3)
O1—C9—C4—C5	106.4 (4)	N3—C9—C8—N4	28.3 (3)
N3—C9—C4—C5	−132.8 (3)	C4—C9—C8—N4	−90.0 (3)
C8—C9—C4—C5	−21.4 (4)	O1—C9—C8—C7	−98.1 (4)
O1—C9—C4—C3	−69.0 (4)	N3—C9—C8—C7	146.8 (3)
N3—C9—C4—C3	51.9 (4)	C4—C9—C8—C7	28.5 (4)
C8—C9—C4—C3	163.2 (3)	C5—C4—C3—C2	−0.1 (5)
C12—N2—C6—C7	2.5 (5)	C9—C4—C3—C2	175.4 (3)
C12—N2—C6—C5	−178.3 (3)	C9—N3—C13—N4	8.9 (4)
C10—C7—C6—N2	−3.1 (5)	C9—N3—C13—S1	−169.8 (3)
C8—C7—C6—N2	179.9 (3)	C8—N4—C13—N3	12.8 (4)
C10—C7—C6—C5	177.7 (3)	C8—N4—C13—S1	−168.6 (3)
C8—C7—C6—C5	0.7 (5)	C6—C7—C10—C11	0.7 (6)
N1—C5—C6—N2	8.0 (5)	C8—C7—C10—C11	177.7 (4)
C4—C5—C6—N2	−171.1 (3)	C4—C3—C2—C1	0.8 (5)
N1—C5—C6—C7	−172.7 (3)	C5—N1—C1—C2	1.1 (6)
C4—C5—C6—C7	8.1 (5)	C3—C2—C1—N1	−1.3 (6)
C13—N4—C8—O2	91.6 (4)	C6—N2—C12—C11	0.6 (6)
C13—N4—C8—C7	−149.3 (3)	N2—C12—C11—C10	−2.8 (6)
C13—N4—C8—C9	−26.5 (4)	C7—C10—C11—C12	2.1 (6)
C6—C7—C8—O2	−145.7 (3)		

### Hydrogen-bond geometry (Å, °)

**Table:**

*D*—H···*A*	*D*—H	H···*A*	*D*···*A*	*D*—H···*A*
N3—H3N···O1^i^	0.93 (4)	2.08 (4)	2.980 (4)	162 (3)
O2—H2O···S1^ii^	0.82	2.49	3.276 (3)	160.
N4—H4N···S1^iii^	0.90 (4)	2.48 (4)	3.365 (4)	166 (3)
O1—H1O···N1^iv^	0.82	2.33	2.930 (4)	130.
O1—H1O···N2^iv^	0.82	2.26	3.032 (4)	158.

Symmetry codes: (i) *x*, −*y*+3/2, *z*+1/2; (ii) *x*, −*y*+3/2, *z*−1/2; (iii) −*x*+1, −*y*+1, −*z*+1; (iv) −*x*, −*y*+1, −*z*.
